# Perspectives and Recommendations Regarding Standards for Ultraviolet-C Whole-Room Disinfection in Healthcare

**DOI:** 10.6028/jres.126.015

**Published:** 2021-08-20

**Authors:** Arthur Kreitenberg, Richard A. Martinello

**Affiliations:** 1 University of California Irvine School of Medicine, Irvine, CA 92617, USA; 2 Yale School of Medicine and Yale New Haven Health, New Haven, CT 06510, USA

**Keywords:** hospital rooms, infection, ultraviolet-C, whole-room disinfection

## Abstract

Patient well-being must be the driving force for determining standards for disinfection systems based on ultraviolet-C (UV-C) radiation. Reductions of inoculated bacteria on carriers is the optimal method of validating a UV-C–emitting system. We make specific, evidence-based recommendations regarding room description, organism selection, carrier material, quantity, orientations, and locations. Criteria for a satisfactory performance are discussed. Adoption of these requirements will ensure that devices intended for room disinfection provide the greatest chances for prevention of environmentally derived healthcare-associated infections

## 1 Introduction and Motivation

The use of ultraviolet (UV) radiation for disinfection is not uncommon. It is well understood that UV radiation inactivates microorganisms, including viruses, via degradation of their genetic material and inhibition of their ability to reproduce. Moreover, it is well understood that the ultraviolet-C (UV-C) band (200 nm–280 nm) is most commonly used for disinfection [1]. UV-C in the range of 254 nm has been shown to reduce bacteria on surfaces, with the extent of reduction depending on the device, cycle time, and type of pathogen [2, and additional references within], and this is the focus of this perspectives paper.

Healthcare administrators, infection-control professionals, epidemiologists, public health experts, nurses, and physicians are charged with selecting products and methods that effectively disinfect the healthcare environment to achieve a decrease in risk for healthcare-associated infections (HAIs). The U.S. Food and Drug Administration (FDA) 21 CFR Section 888.6600 [3] regulates UV chambers but specifically exempts whole-room devices. This leaves a regulatory void and underscores the need for reliable and reproducible standards detailing the laboratory testing and analytical protocols to be used in assessing the effectiveness and efficacy of UV disinfection devices. Few studies [4, 5] published in the peer-reviewed medical literature have considered the impact of basic physical realities affecting the use of UV-C radiation for surface disinfection such as time, distance, angles, and shadowing. Few studies showing impact on HAIs have used randomized, controlled, interventional investigations [6, 7]. To guide both industry and investigators, rigorous, reliable, and meaningful efficacy testing standards are needed for user and patient safety to ensure that a device being purchased meets a benchmark of killing germs, so users may make informed decisions that impact patients’ well-being.

The U.S. Department of Health and Human Services Center for Medicare & Medicaid Services imposes a 1% payment reduction penalty on hospitals with a total Hospital Acquired Condition score in the worst-performing quartile, including infections [7]. Ultimately, however, it is the patient that pays the highest price when an HAI strikes.

In the United States, the measurement of HAI rates is generally performed using detailed case definitions that have been standardized by the U.S. Centers for Disease Control and Prevention (CDC) [8]. However, numerous factors and variables, many of which are difficult to observe, let alone measure, present a challenge in the efforts to prevent HAIs. Studies seeking to show a reduced infection rate due to a specific intervention typically require exceedingly high subject numbers to reach statistical validity. However, reduction of environmental biological burden is relatively easy to measure. While only a proportion of all HAIs is related to environmental contamination, standards should be appropriately based on reduction of pathogens in the environment, because fewer pathogens would be expected to cause fewer HAIs [9].

In this discussion, we advocate for the highest possible fidelity to real-world healthcare environments in testing. The establishment of industry testing standards would be instrumental in this regard. While patient needs must dictate the standards, it is expected that the development and implementation of such standards will ultimately also help industry, users in healthcare, and investigators researching UV-C use in healthcare. The shared objective is to accelerate the establishment of clarity for device effectiveness and use.

## 2 Culture-Based Standards Versus Ultraviolet Metrology/Dosimetry/ Radiometry

Dead pathogens do not cause infections. To confirm true microbe deactivation, only culture-based methods can be relied upon to protect patients. Biological burden determination based on quantitative adenosine triphosphate (ATP) cannot distinguish living from dead germs. Emerging technologies that seek to replace environmental cultures must correlate data with infectious potential. Disparity between dosimetry and actual germ-kill rate may be compounded by the inherent limitations of UV-C with regard to distance, angle of incidence, and shadowing, as well as the effect of texture of the target surface.

Germ-kill, or viral deactivation, is commonly measured as percent reduction in surviving microbes. For example, if the test surface initially contains 15,000 bacteria, and the intervention reduces this number to 1500, then there has been a 90% reduction. If the intervention reduces the number to 15, then there has been a 99.9% reduction. A mathematical shorthand convention expresses these relative to a logarithmic base 10 value. In these examples, a 90% reduction is a 1 log_10_ reduction, and a 99.9% reduction is a 3 log_10_ reduction. For simplicity of conversion, simply count the number of nines to arrive at the log_10_ reduction. A 99.9999% reduction, using this shorthand, is shown as a 6 log_10_ reduction.

Accurate measurement of UV-C dosing, typically measured in millijoules per square centimeter (mJ/cm^2^), may predict actual germ-kill rates. However, high-quality radiometers are expensive, complex, and require calibration. Paper dosimetry [4] is cheap, fast, and simple, but accuracy, precision, reliability, and reproducibility problems limit its use to screening only.

Evolving UV-C technologies, including excimer lasers, light-emitting diodes (LEDs), and pulsed xenon lamps, may produce a multitude of wavelengths within the germicidal ultraviolet spectrum. UV-C meters are generally filtered to a specific wavelength and may not accurately measure far-UV-C or broad-spectrum output covering many germicidal wavelengths. Varying wavelengths have different efficiencies at reducing microbial contamination [10]. A standard determined by culture-based germicidal activity independent of a specific UV-C wavelength can level the playing field for current and future technologies.

There are hundreds of studies [10] demonstrating individual microbe susceptibilities and logarithmic reductions to UV-C dosing, generally at 254 nm. However, the UV-C dosing values used to obtain a given reduction are quite variable [3]. This is likely due to nonstandardization of methodologies, including microbe density, soil load, measuring techniques, and aqueous, air, or surface environmental conditions. There is a great need for standardization of measurement techniques and UV-C application to develop a reliable understanding of the dose needed to deactivate specific microbial genera and species as well as strain variants within a species. Until then, these studies are guidelines at best, and culture-based testing remains the “gold standard.”

Once a device and method have been shown to be effective by a culture-based standard, output of an individual UV-C device may be monitored with calibrated and reliable UV-C metrology.

## 3 Criteria and Recommendations

### 3.1 Hospital Rooms

Since hospital rooms are the primary intended site for UV-C surface disinfection, ideally, the testing agency should use a mockup or an actual patient room for UV-C device validation. Hospitals may understandably reject having potential pathogens intentionally introduced into their facility, even on carriers, leading to the use of hospital room mockups for testing rather than active hospital rooms. A hospital room mockup should represent an actual hospital room with extremely high fidelity, including the room’s dimensions, surfaces, equipment, and furnishings. This is important because the variety of surfaces commonly used in healthcare settings should be directly tested. A real-world setting can mimic the shadowing and UV-C reflectiveness typical to a hospital patient room.

Specific room equipment/furnishings must include, at a minimum, a patient bed with bedrails, controls, and nurse-call button, overbed table, nightstand, intravenous drip pole with infusion pump, visitor chair, and bedside commode. Cardboard cutouts of these furnishings are acceptable if they are dimensionally accurate. Providing real-world furnishings increases the fidelity of the simulation and assures end users of the standard’s validity.

The hospital room mockup must include an adjoining bathroom with a shower enclosure, toilet, and countertop/sink with valve handles. A simulated hospital room without a simulated bathroom will not benefit patients. UV-C from a room-based emitter is “line-of-sight” and will not “bounce off” the walls into the bathroom for effective disinfection.

Ceiling-mounted hospital privacy curtains surrounding the bed are ubiquitous in healthcare and must be included in any simulated hospital room. Curtains often harbor HAI pathogens [11]; when the curtain is folded and stowed, only about 25% of the curtain surface is exposed to UV-C. When the curtain is unfolded and stretched around the bed, UV-C is blocked from the remainder of the room. Failure to include curtains in the hospital room mockup will result in false assurances of efficacy in practice. Efficacy of UV-C on fabrics depends on the material used, the texture, and absorbency of the material.

Intensive care units (ICUs) represent a different kind of hospital room, with different dimensions, enclosures, and furnishings. Consideration may be given to an appropriate ICU mockup, although it will likely share most of the criteria of a hospital room.

The operating room is a specialized hospital room distinctly different in many ways from a hospital patient room. *Clostridiodes difficile* is not typically transmitted in the operating room. *Staphylococcus*, *Streptococcus*, *Pseudomonas*, and *Escherichia coli* represent the majority of surgical-site infections acquired in the operating room. These organisms are generally much more susceptible to ultraviolet irradiation than is *Clostridiodes difficile*. However, a higher disinfection level is required in an operating room than a patient room, and a standard should reflect this.

The furnishings within an operating room are quite different than those in a patient room. There are neither curtains nor bathrooms. Surfaces tend to be hard, including tile, glass, stainless steel, and plastic. In addition, there are over 5000 free-standing ambulatory surgery centers in the United States, where a simple hospital room standard would be meaningless to such facilities when making purchasing decisions. Moreover, operating room disinfection is extremely time-sensitive, so any standard must include a standard for disinfection time interval.

### 3.2 Organism

*Clostridiodes difficile* is the test organism of choice because it is considered to be the most UV-C resistant of HAI-causative organisms. There is no universally agreed UV-C dose to reduce this organism on surfaces, so UV-C measurement cannot be used as a standard. In contrast, *Clostridiodes difficile* cultures are straightforward, reproducible, and reliable.

### 3.3 Nature of the Carrier

A “carrier” is a small standardized sample or swatch of material to be inoculated, exposed to UV-C and then cultured so as to reliably and reproducibly test disinfection methods. Smooth stainless-steel carriers are common in the research setting, but in practice, outside of the operating room and some specialty areas, few commonly touched hospital-room surfaces are made of smooth stainless steel. Differences between UV-C effectiveness and germ survival on stainless steel versus laminates, plastic, vinyl, linoleum, wood, and other common materials are not quantified in the literature. To assure real-world fidelity, laminate carriers should be used for laminate countertops, vinyl/linoleum carriers should used for vinyl/linoleum flooring, and plastic carriers should used for plastic surfaces, *etc*.

The texture of the carriers should reflect textures commonly used in hospitals. Texture is important for two reasons. First, textured surfaces have a greater effective surface area than smooth surfaces. Since UV-C delivery is measured in light energy per unit surface area, increased light energy must be delivered to an increased surface area to be effective. Second, consider a textured surface with “valley” depths of 100 μm, about the diameter of a human hair. Bacteria measure about 1 μm, and viruses are about 0.1 μm. Pathogens may “hide” in the valleys at a depth equivalent to a person standing in a 2000 m deep canyon. UV-C lamps that are placed off to the side of a horizontal surface like a bed, table, or countertop cannot reach into the surface’s valley floors. By contrast, UV-C lamps that are placed directly above the horizontal surface can reach the microbes in the valley floors. This overhead effect may be enhanced by motion of the lamps like the sun moving over a canyon. This has been described as the “canyon wall effect” by Jaffe [12].

Cadnum *et al*. [13] studied *Clostridiodes difficile* reductions on different materials with UV-C lamps arranged parallel to the test carrier surface. Stainless steel had a 1.5 log_10_ reduction, whereas Formica® had a 1.0 log_10_ reduction at the same UV dose. This means the organism had more than triple the survival rate on Formica as compared to stainless steel. Because of the canyon wall effect of textured Formica, the differences would likely be far greater when UV-C is applied from the side of a horizontal textured surface.

### 3.4 Carrier Quantity and Orientation

The U.S. Environmental Protection Agency (EPA) standard for hospital and healthcare chemical disinfectants [14] utilizes 60 carriers for each of two different bacteria types contaminated with 6 to 7 log_10_ bacteria and showing complete reductions in 10 min for at least 57 of 60 carriers for *Staphylococcus aureus* and 50 of 60 carriers for *Pseudomonas aeruginosa*. The EPA standard does not apply to, nor is it practical for, UV-C. Although 120 carriers minimize the risk of sampling error, placing 120 carriers within a high-fidelity simulated patient room is overburdensome and costly. Placing too few carriers in a room may lead to serendipitous or strategic placement of a UV-C device and give false assurance of widespread room disinfection.

Therefore, we recommend that future standards specify 50 carriers placed throughout the simulated patient room and bathroom spread widely and on a multitude of surfaces as described in the following section. Pathogens may be present on any commonly touched surface. If 50 widely spread, high-touch locations in a single room are shown to reduce germ load, it is likely the same is true for the entire room. Use of many carriers widely spread will minimize sampling error caused by too few data points.

UV-C effectiveness is greatly affected by the angle of incidence between the UV-C source and the target surface [3]. Therefore, carriers should be placed horizontally onto a horizontal surface and attached vertically onto a vertical surface.

### 3.5 Key Carrier Locations

It is reasonable to set a UV-C device standard to disinfect a wide array of surfaces throughout the room and bathroom to ensure optimal patient safety. Jefferson *et al*. [15] found that about 50% of high-touch surfaces in the operating room are missed by crews using chemical disinfection. Surgical cleaning staff tend to be more specialized and trained, and operating rooms are treated several times per day. It can be safely assumed that similar or worse coverage occurs in regular patient rooms.

#### 3.5.1 Floor

Airborne pathogens tend to settle preferentially onto horizontal rather than vertical surfaces [16]. The largest horizontal surface in any room is the floor. Orthopedic operating room contamination has been shown to increase with room “traffic” as measured by door openings [17]. Personnel presence and movement, with mechanical and air-flow turbulence, are the likely causes of re-aerosolization of pathogens from surfaces. The airborne pathogen can then land onto the surgical site. Therefore, it is recommended that at least 8 carriers be placed about the floor. Because UV-C emitters are likely to be placed over floor locations commonly walked over, at least one carrier should be placed beneath the UV-C emitting machine in each of its locations.

#### 3.5.2 Furnishings

Multiple carriers should be placed on the furnishings, with an emphasis on commonly touched surfaces, such as the undersurface edge of the patient’s overbed table, where it is typically grasped. The carrier should be placed horizontally on a horizontal surface and attached vertically to a vertical surface. Each relevant surface of every piece of furniture should have a carrier attached. Positions of knobs and handles should have separate carriers. Doorknobs, assistance call buttons, television controls, light switches, and other commonly touched surfaces in the room all warrant carriers.

#### 3.5.3 Curtains

Hospital privacy curtains are frequently touched and have been demonstrated to be contaminated with HAI pathogens [11]. Both sides of the curtain at the leading and trailing edges as well as the midpoint of the curtain at approximately shoulder to elbow level should have carriers attached. These locations are where the curtain is most commonly touched.

#### 3.5.4 Bathrooms

Commonly touched surfaces in the bathroom, including doorknobs, light switches, sink valve handles, shower controls, toilet flush levers, and sides of the toilet seat, should all have carriers to monitor disinfection efficiencies. Horizontal surfaces require horizontal carriers, and vertical surfaces require vertical carriers.

#### 3.5.5 UV-C Machine Wheels and Power Cords

Jencson *et al*. [18] demonstrated that wheelchair wheels in the hospital are contaminated with HAI-causative organisms. UV-C machines are preferentially deployed in rooms likely to be contaminated. These machines have the potential of cross-contaminating rooms via their wheels and power cords. Because placing carriers around the outer wheel circumference precludes repositioning of the UV-C emitter device, carriers should be placed on the floor beneath the machine and adjacent to the wheel. Similarly, power cords can cross-contaminate successive rooms and block UV-C from reaching the floor beneath the cords. A carrier should be placed adjacent to the power cord where it touches the floor near the wall electrical outlet.

## 4 Qualifications of UV-C Machines

Manufacturers should be encouraged to innovate and should be given a fair opportunity to meet patient-centric standards. Manufacturers should supply or designate the operator to assure that the manufacturer’s directions for device use are strictly followed. Operators should be shown exactly where the 50 carriers are located, and no carriers should be hidden. Operators should be apprised of the approval criteria prior to the testing. They may be allowed as much time as desired. Time should be recorded by personnel or the machine, from insertion into the room until the room is vacated.

A minimum of 45 (90%) of the carriers should have at least a 3 log_10_ reduction of *Clostridiodes difficile.* The remainder should have at least a 2 log_10_ reduction. The rationale is that it only takes one touch to a surface to potentially cause an HAI. Time will be recorded but should not be a criterion for passing. A confirmatory trial should be repeated on at least one additional day.

Although these standards may appear draconian, they should be considered in relation to the human and financial costs of a single HAI. Patients deserve both a reliable test standard and assurance of legitimacy when they are told their room has been disinfected.
